# On the Relations and Differences between Gout and Scrofula, with Reference Especially to Consumption

**Published:** 1839-07

**Authors:** 


ART. V.—*Over de Overeenkomst en het Verschil Tusschen de Jicht en de Scrophulosis, vooral met Betrekking tot de Longetering*; *eene Voorlezing door* A. A. SEBASTIAN, M.D., Hoogleeraar in de Geneeskunde aan de Hoogeschool te Groningen.—*Groningen*, 1838.

*On the Relations and Differences between Gout and Scrofula, with reference especially to Consumption.* By A. A. SEBASTIAN, Professor of the Medical Faculty of the University of Groningen.—*Groningen*, 1838. 8vo, pp. 102.

THE chief object of this short treatise is to prove that gout and scrofula are in many points very closely allied to each other, or rather, perhaps, to show that they are mere modifications of the same diseased state. They both depend, according to our author, upon imperfect assimilation of the food, and the one occasionally passes in an imperceptible manner into the other, so that a subject who has been affected with scrofula in early life is extremely apt to suffer from gout in old age. The symptoms of the two diseases are to a certain extent alike; in both, the same tissues and organs are liable to be attacked, as the eye, the skin, the joints, the serous membranes, &c.; and both occur most frequently in the same localities.

Consumption depends upon internal scrofula, and consequently is also allied to gout. Tubercles and gouty concretions are composed alike of animal and earthy substances; and the former are occasionally found to contain uric acid as well as the latter. Thus, according to Sebastian, scrofula, gout, and consumption, are mere modifications of each other. A scrofulous patient, under favorable circumstances, will suffer from gout only, when in less favorable circumstances he would have fallen a victim to phthisis. In the children of gouty subjects the hereditary taint is often changed from that of gout to that of phthisis; and the latter disease makes its appearance accordingly.

Such are the leading features of the treatise before us. It is a statement of general doctrines not sufficiently supported by evidence; but we think the author's intention is more to direct notice to a subject of considerable practical importance than to make any important additions to our previous knowledge of the diseases in question.

